# Optimizing Photodynamic Therapy for Cervical Esophageal Cancer: A Novel Technique for Precise Lesion Targeting by Transnasal Thin Endoscopy and Upward-Facing Attachment

**DOI:** 10.7759/cureus.81238

**Published:** 2025-03-26

**Authors:** Tomoyuki Hayashi, Masaki Nishitani, Masaki Miyazawa, Akihiro Seki, Hidetoshi Nakagawa, Kouki Nio, Takeshi Terashima, Noriho Iida, Shinya Yamada, Hajime Takatori, Tetsuro Shimakami, Taro Yamashita

**Affiliations:** 1 Gastroenterology, Kanazawa University, Kanazawa, JPN

**Keywords:** cervical esophageal cancer, chemoradiotherapy, esophageal cancer, photodynamic therapy, talaporfin sodium

## Abstract

Photodynamic therapy (PDT) with talaporfin sodium shows promise as a salvage treatment for locoregional recurrence of esophageal cancer after chemoradiotherapy (CRT). However, its application in cervical esophageal cancer is limited due to anatomical challenges, such as the restricted maneuverability of standard endoscopes and difficulty in achieving stable and perpendicular laser irradiation.

This study introduces a modified PDT technique using a thin endoscope with a customized attachment for lesion stabilization and precise laser targeting. The attachment, wrapped in black plastic tape, facilitated perpendicular irradiation and reduced laser scattering.

Two cases of recurrent cervical esophageal cancer after CRT were successfully treated with this method. The first patient had a 10-mm, 0-IIc+IIa, cT1b lesion with severe stenosis, necessitating the use of a thin endoscope. PDT was performed with 600 J irradiation, achieving complete local control without complications, with no recurrence over four years. The second patient had a 20-mm, 0-IIa, cT1b lesion and was treated with 800 J irradiation, resulting in complete local control within three months and no recurrence at one year.

Despite these promising results, this modified PDT technique has several limitations. The small sample size (two cases) limits generalizability, and long-term outcomes beyond the current follow-up period remain unknown. Additionally, the technique requires specialized equipment and expertise, potentially limiting its widespread adoption. This study presents a novel modification of PDT using transnasal thin endoscopy for precise targeting of cervical esophageal cancer lesions. This approach minimizes laser scattering and enhances procedural accuracy. We report two cases with complete local control and no recurrence over one to four years. While promising, further studies are required to assess its long-term efficacy and broader applicability.

## Introduction

Esophageal cancer is the eleventh most prevalent malignancy and the seventh leading cause of cancer-related mortality, with more than 445,000 deaths annually [1]. Definitive chemoradiotherapy (CRT) and radiotherapy (RT) are the primary treatment options, although locoregional recurrence remains a major concern, with failure rates ranging from 50% to 55% [2,3]. In these cases, salvage surgery is often required, despite its association with high complication (50%-77%) and mortality rates (approximately 15%) [4-7]. For patients with locoregional failure after RT without distant metastases or lymph node involvement, local treatments, such as photodynamic therapy (PDT), may be considered, particularly when endoscopic resection is not feasible [8-10]. PDT works by activating a photosensitizer within cancerous tissues using a specific wavelength of light, which induces a photochemical reaction that results in the destruction of cancer cells and triggers acute inflammation [11,12]. PDT is a well-established and effective treatment for various cancers, including those of the gastrointestinal tract [13-16].

Talaporfin sodium (Laserphyrin®; Meiji Seika Pharma Co., Ltd., Tokyo, Japan), a second-generation photosensitizer, is quickly cleared from the skin and necessitates only a brief sunshade period (≤2 weeks) [17,18]. PDT with talaporfin sodium is a promising salvage treatment for locoregional failure after CRT in patients with esophageal cancer. A phase II multicenter study of 28 histologically confirmed lesions demonstrated a high local complete response rate of 88.5% (T1: 100%; T2: 57.1%) [19]. This innovative approach is being gradually incorporated into clinical practice in Japan.

PDT is not typically recommended for cervical esophageal lesions due to significant challenges in achieving precise targeting. These challenges include the risk of unintended laser exposure to the larynx caused by endoscope displacement and difficulty in accurately irradiating lesions at sites of physiological stenosis within the cervical esophagus. This makes standard PDT less effective for lesions located in the cervical region. However, given the limited treatment options available following CRT or RT, there is a high demand for alternative therapies such as PDT for cervical esophageal cancer.

The local control rate for cervical lesions using standard PDT has been reported to be significantly lower than for non-cervical lesions [20]. To address these limitations, we developed a modified PDT technique specifically designed for treating difficult-to-reach cervical esophageal cancer. The modification aims to reduce the risks associated with laser scattering and improve the accuracy of irradiation despite anatomical constraints.

In this study, we present two cases treated with this novel approach and describe the modifications implemented to overcome the anatomical challenges of cervical lesions. It aims to evaluate whether a modified PDT technique using thin endoscopy and an upward-facing attachment improves lesion targeting and treatment outcomes compared to conventional PDT approaches. We hypothesize that this modified PDT technique will provide more effective local control and reduce complications in treating cervical esophageal cancer compared to standard methods. Our research question is whether this modification can improve the clinical outcomes of PDT in cervical esophageal cancer, particularly in cases previously considered too difficult for standard PDT.

## Case presentation

The cervical esophageal cavity has a very narrow working space, owing to physiological narrowing, and the frequent gag reflex makes it difficult to accurately irradiate forward while keeping the scope fixed. Additionally, the scope is prone to contact the lesion, making it difficult to ensure a clear field of view.

We found that when treating cervical esophageal cancer with a thin endoscopic fiber, it provides greater stability and allows for more effective PDT compared to a transoral endoscopic fiber. This is because the probe is thin enough to pass through the forceps channel of the endoscope.

**Figure 1:**
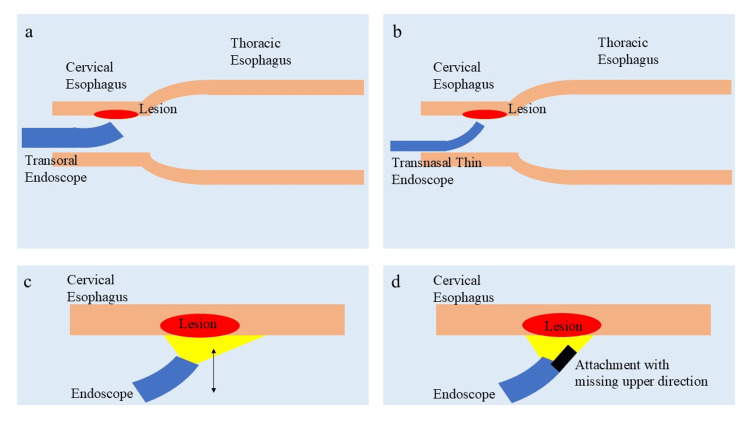
Optimized photodynamic therapy for cervical esophageal cancer. a: The cervical esophageal cavity has a very narrow working space due to physiological constriction, and the frequent gag reflex makes it challenging to approach the lesion vertically. b: A thin transnasal endoscopic fiber provides greater stability, making it easier to approach the lesion vertically. c: Due to the narrow working space, the scope tends to come into contact with the lesion, making it difficult to maintain a clear field of view and increasing the risk of laser light scattering. d: A soft attachment with a missing upper section was added to the tip of the scope, facilitating vertical access to the lesion without direct contact. Additionally, the attachment was wrapped in black vinyl tape to prevent laser light from scattering in other directions. This figure has been created by the authors.

Additionally, a soft attachment was attached to the tip of the scope. The attachment was designed without an upper section, allowing for easier vertical access to the lesion. Furthermore, the attachment was wrapped in black plastic tape to prevent the laser from scattering in other directions (Figure 1).

Before using this special attachment, we obtained approval from our hospital department to manage highly complex new medical technologies.

Two consecutive cases of successful irradiation are presented since these attempts began. Successful treatment was defined as the absence of residual disease or recurrence on endoscopic examination three months after treatment, with confirmation of local control.

Case 1

*Patient Presentation* A woman in her 60s without symptoms was diagnosed with hypopharyngeal cancer (Stage IV A) and midthoracic esophageal cancer (Stage I) in March 2020. She underwent intra-arterial chemotherapy consisting of four cycles of cisplatin for hypopharyngeal cancer, followed by CRT for esophageal cancer, which included two cycles of 5-fluorouracil and cisplatin, along with 60 Gy of radiation covering the entire esophagus, including the cervical esophagus. This treatment resulted in a complete response.

*Treatment Decision* In June 2023, a new carcinoma, distinct from the primary lesion, was detected in the cervical esophagus. Due to severe radiation-induced stenosis, the passage of a conventional oral endoscope was blocked, and a thin endoscope was necessary for examination; hence, PDT was chosen as the salvage therapy.

**Figure 2:**
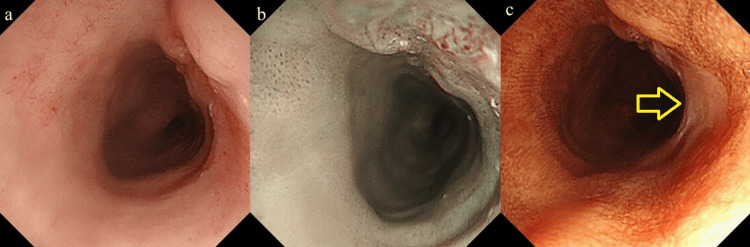
Endoscopic image of case 1 before PDT. a: A 10-mm, 0-IIc+IIa, T1b lesion was identified in the cervical esophagus, located 17 cm from the incisor. This lesion was positioned 2 cm from the pharyngoesophageal junction, which is critical for understanding its relationship to the surrounding anatomy and treatment approach. b: Narrow-band imaging (NBI) revealed a brownish area with dilated intraepithelial papillary capillary loops. Due to the use of a transnasal endoscope, magnified observation was not possible, limiting the ability to fully evaluate microvascular patterns. The NBI provided insight into vascular irregularities within the lesion. c: Lugol's staining identified an adjacent 0-IIb lesion (arrow), consequently, the tumor size became approximately 25 mm. This lesion appeared distinct from the main lesion, and Lugol’s staining helped define its margins and evaluate the extent of mucosal involvement. PDT: Photodynamic therapy.

*Procedure Details* Endoscopic examination revealed severe radiation-induced stenosis of the pharynx, necessitating a thin transnasal endoscope for examination. The lesion, measuring 10 mm, 0-IIc+IIa, cT1b, was located 17 cm from the incisor (2 cm from the pharyngoesophageal junction). Narrow-band imaging revealed a brownish area with dilated intraepithelial papillary capillary loops. Lugol's staining identified an adjacent 0-IIb lesion, consequently, the tumor size became approximately 25 mm (Figure 2). The lesion was approached perpendicularly with a thin endoscope (EG-6500N; Fujifilm, Tokyo, Japan), and total of 600 J energy was applied in two (upward and downward) directions. No immediate complications were noted.

**Figure 3:**
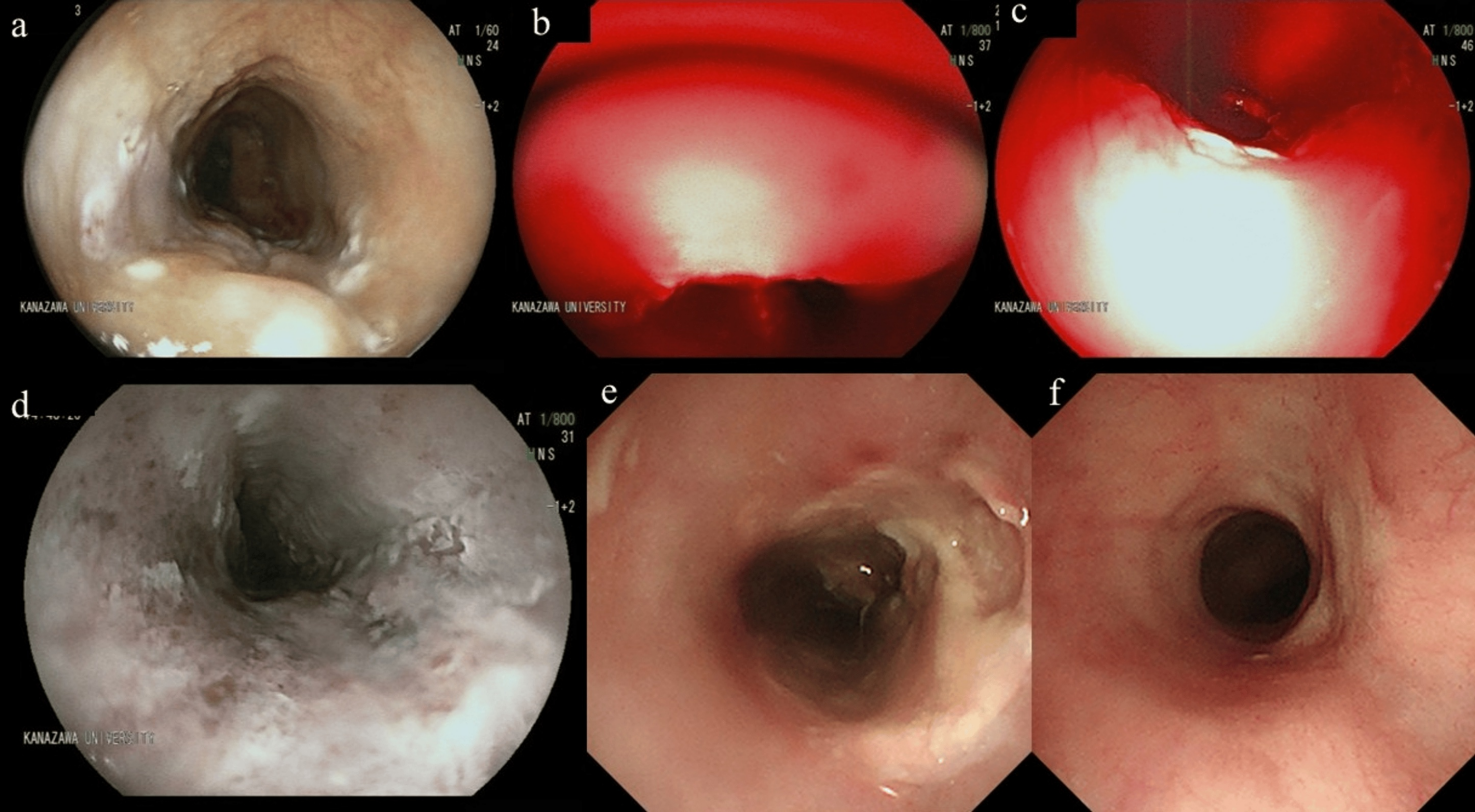
Endoscopic images during and after PDT in case 1. a: After marking around the lesion. b, c: A thin transnasal endoscope was able to approach the lesion more perpendicularly, ensuring stable manipulation. A total of 600 J was irradiated from both upward (b) and downward (c) directions. d: On the following day, uniform ischemic changes were noted within the marked area. e: One week later, the lesions were uniformly ulcerated. f: Three months later, endoscopy confirmed local control without significant complications. PDT: Photodynamic therapy.

*Follow-Up and Outcome* Three months after PDT, endoscopy confirmed complete local control with no significant complications (Figure 3). No local recurrence was observed over a follow-up period of four years. The patient did not report any significant changes in swallowing function or quality of life during the follow-up period.

Case 2

*Patient Presentation* A man in his 60s without symptoms was diagnosed with cervical and mid-thoracic esophageal cancer in February 2021 and underwent CRT (two cycles of 5-fluorouracil and cisplatin plus 60 Gy of radiation), resulting in a complete response.

*Treatment Decision* In November 2023, a lesion was detected in the cervical esophagus, suggestive of local recurrence. Due to difficulty maneuvering a conventional oral scope, PDT was chosen as a salvage treatment, and approval for the procedure was obtained from the hospital’s high-difficulty medical technology management division.

**Figure 4:**
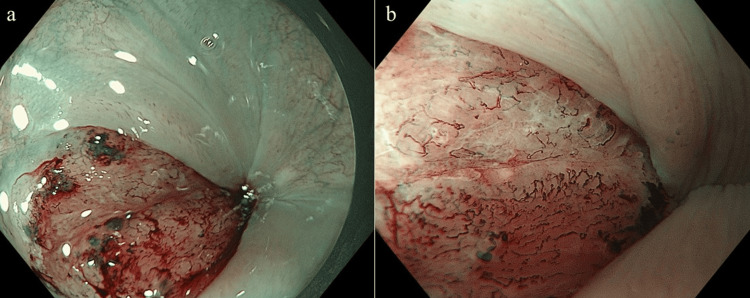
Endoscopic image of case 2 before PDT. a: Endoscopic image showing a 20-mm, 0-IIa, cT1b lesion located 18 cm from the incisors in the cervical esophagus. The lesion was identified based on its size, shape, and location within the esophagus. b: Narrow-band imaging revealing disrupted intraepithelial papillary capillary loops in the lesion. The lesion was classified as Type B2 according to the Japanese Esophageal Society’s classification. PDT: Photodynamic therapy.

*Procedure Details* The lesion, measuring 20 mm, 0-IIa, cT1b, was located 18 cm from the incisor (2 cm from the pharyngoesophageal junction). The lesion was classified as Type B2 according to the Japanese Esophageal Society classification system (Figure 4). A thin endoscope (GIF-H190N; Olympus Medical Systems, Tokyo, Japan) with a customized attachment was used to facilitate better maneuverability and proper distance from the lesion. PDT was successfully performed with no major complications, and a total energy dose of 800 J energy was applied.

**Figure 5:**
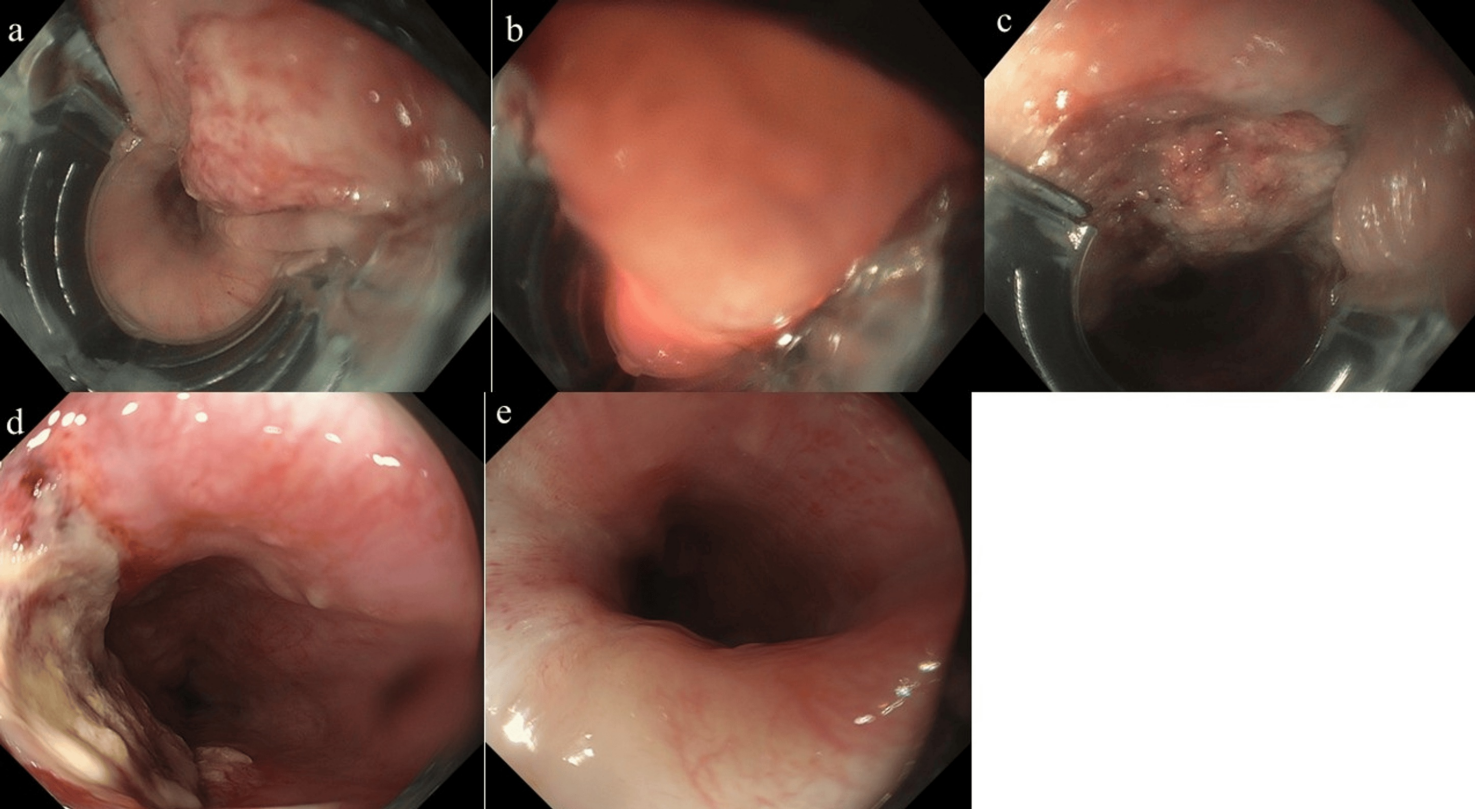
Endoscopic images during and after PDT in case 2. a: A transoral endoscope attachment was modified by removing its upper section to fit a thin endoscope. b: The attachment allowed for better maneuverability and a proper distance from the lesion. c: On the following day, uniform ischemic changes were noted within the elevated lesion. d: One week later, the lesions were uniformly ulcerated. e: Local control was confirmed at 3 months after treatment. PDT: Photodynamic therapy.

*Follow-Up and Outcome* Three months after treatment, endoscopy confirmed local control without recurrence (Figure 5). The patient did not experience any major side effects, and there was no local recurrence at one year. Long-term effects on swallowing function and quality of life were not reported in detail, but the patient did not experience significant issues during follow-up.

## Discussion

Compared to other salvage treatments after CRT, such as surgery or endoscopic resection, PDT offers a less invasive alternative with the potential for fewer complications. Surgery, although effective in resecting lesions, carries risks of significant morbidity and is often not an option for patients with poor performance status or those who have undergone prior treatments such as CRT. Endoscopic resection is not always feasible due to the fragility and fibrosis of the esophagus after CRT.

PDT has emerged as a promising modality, albeit with limitations in treating cervical esophageal lesions due to anatomical and procedural challenges. Traditional PDT has been shown to have lower efficacy for cervical lesions due to the anatomical challenges involved. In this study, we developed a novel PDT technique specifically tailored for cervical esophageal cancer and demonstrated its feasibility and effectiveness in two challenging cases.

The cervical esophagus presents unique challenges for PDT, including limited working space, frequent gag reflexes, and a heightened risk of endoscope dislodgement. Our approach, which incorporated a thin endoscopic fiber and specially designed attachment, successfully mitigated these challenges. Thin endoscopy provides a stable and precise platform for laser irradiation, while the upward-facing attachment, wrapped in black plastic tape, minimizes laser scattering and ensures accurate targeting of the lesions.

The outcomes of these cases underscore the potential of the modified PDT technique as a salvage therapy for cervical esophageal cancer. The novelty of this approach lies in its adaptability to the cervical esophagus, an area where conventional PDT techniques are often impractical.

In our previous study, we performed PDT on five lesions in the cervical esophagus and achieved a local CR rate of only 20% [20]. Subsequently, we modified the scope and attachment, which enabled successful local control in two consecutive cases. This represents a significant advancement in PDT for cervical esophageal cancer.

Additionally, esophageal stricture is a common postoperative complication of cervical esophageal cancer and is observed in 60% of the cases. However, by limiting the irradiation field, as demonstrated in the present case, no postoperative stricture symptoms were observed in these two consecutive cases.

Furthermore, while conventional laser irradiation of the cervical esophagus has raised concerns about fatal complications, such as postoperative laryngeal edema, our investigations have thus far reported no major complications. This finding suggests that the procedure is both effective and safe.

Despite these promising results, the limitations of this study warrant further discussion. First, it involved only two cases; therefore, larger studies are needed to validate our findings. Second, the long-term safety and efficacy of PDT for cervical esophageal lesions remain uncertain, particularly regarding potential delayed complications. Finally, as this technique requires specialized equipment and expertise, its generalizability may be limited.

Compared to salvage esophagectomy, which carries a high risk of morbidity and mortality (50%-77% complication rates), this modified PDT technique offers a minimally invasive alternative with promising local control rates. However, given the small sample size of this study, further clinical trials are needed to confirm its efficacy and safety in broader patient populations.

## Conclusions

Our modified PDT technique, using transnasal thin endoscopy with an upward-facing attachment, provides a promising and effective salvage therapy for cervical esophageal cancer. This approach improves procedural stability and targeting accuracy, effectively addressing the challenges commonly encountered with conventional PDT in this anatomical region. However, further research is required to establish its long-term safety, efficacy, and broader clinical applicability because of the lack of statistical validation. We propose conducting prospective studies with larger cohorts and extended follow-up periods to validate the effectiveness and safety of this technique across diverse patient populations.
